# Nodal Disease and Survival in Oral Cancer: Is Occult Metastasis a Burden Factor Compared to Preoperatively Nodal Positive Neck?

**DOI:** 10.3390/cancers14174241

**Published:** 2022-08-31

**Authors:** Selgai Haidari, Katharina Theresa Obermeier, Moritz Kraus, Sven Otto, Florian Andreas Probst, Paris Liokatis

**Affiliations:** 1 Department of Oral and Maxillofacial Surgery and Facial Plastic Surgery, University Hospital LMU Munich, 80337 Munich, Germany; 2 Department of Orthopaedics and Trauma Surgery, Musculoskeletal University Center Munich, University Hospital LMU Munich, 80337 Munich, Germany

**Keywords:** occult metastases, neck dissection, survival, oral squamous cell carcinoma

## Abstract

**Simple Summary:**

Occult metastasis in oral squamous cell carcinoma patients is a feared complication. However, there are barely any existing data on survival of patients suffering from occult metastasis. This study aims to compare patients with oral squamous cell carcinoma, considering survival in occult metastases and different treatment approaches.

**Abstract:**

The impact of neck involvement and occult metastasis (OM) in patients with oral squamous cell carcinoma (OSCC) favors an elective neck dissection. However, there are barely any existing data on survival for patients with OM compared with patients with positive lymph nodes detected preoperatively. This study aims to compare survival curves of patients suffering from lymph nodal metastases in a preoperatively N+ neck with those suffering from OM. In addition, clinical characteristics of the primary tumor were analyzed to predict occult nodal disease. This retrospective cohort study includes patients with an OSCC treated surgically with R0 resection with or without adjuvant chemoradiotherapy between 2010 and 2016. Minimum follow-up was 60 months. Kaplan–Meier analysis was used to compare the survival between patients with and without occult metastases and patients with N+ neck to those with occult metastases. Logistic regression was used to detect potential risk factors for occult metastases. The patient cohort consisted of 226 patients. Occult metastases occurred in 16 of 226 patients. In 53 of 226 patients, neck lymph nodes were described as suspect on CT imaging but had a pN0 neck. Higher tumor grading increased the chance of occurrence of occult metastasis 2.7-fold (OR = 2.68, 95% CI: 1.07–6.7). After 12, 24, 48 and 60 months, 82.3%, 73.8%, 69% and 67% of the N0 patients, respectively, were progression free. In the group with OM occurrence, for the same periods 66.6%, 50%, 33.3% and 33.3% of the patients, respectively, were free of disease. For the same periods, respectively, 81%, 63%, 47% and 43% of the patients in the N+ group but without OM remained disease free. The predictors for progression-free survival were a positive N status (HR = 1.44, 95% CI: 1.08–1.93) and the occurrence of OM (HR = 2.33, 95% CI: 1.17–4.64). The presence of occult metastasis could lead to decreased survival and could be a burdening factor requiring treatment escalation and a more aggressive follow-up than nodal disease detected in the preoperative diagnostic imaging.

## 1. Introduction

Oral squamous cell carcinoma (OSCC) is the most common cancer in the oral cavity [1]. The average 5-year-survival rate is 64.4–79.3% [2]. However, if lymph node metastases appear, the survival rate drops down to 50% [3].

Sensitivities of 64% to 93% for presurgical magnetic resonance imaging (MRI) and of 66% to 85% for presurgical computed tomography (CT) have been reported in the detection of cervical lymph node metastasis [4]. Although, in a meta-analysis from Sun et al., 2022, better sensitivity and specificity was found for the MRI than the CT [5], other studies show similar sensitivity rates for both MRI and CT [6]. Relatively high sensitivity (75% to 90%) and specificity (94% to 99%) are reported in detecting lymph node metastases with 8F-fluorodeoxyglucose positron emission tomography (18FDG-PET) [7]. Still, when PET was studied in clinically node-negative necks only, sensitivity of around 50% was reported [8]. Overall, the diagnostic workup fails to reveal metastasis in 20–25% of the patients [9,10,11].

The risk of occult metastases is related to the tumor stage, among other factors. While the rate of occult metastases in T1 and T2 tumors is about 20–30%, the risk may be higher in stages T3 and T4, partly due to the greater depth of invasion [12]. Depth of invasion is an essential factor in the UICC-staging system and one of the parameters that increases the risk of lymph nodal spreading [13,14]. Other risk factors for occult metastases are histopathological parameters such as lymphovascular invasion [15] and histopathological grading [16]. Furthermore, several gene and marker expression patterns have already been studied as risk factors for occult metastases [17,18].

The impact of the neck involvement on the survival of the patients combined with the relatively high rate of occult metastasis has favored elective neck dissection against the Wait and Watch approach [19]. However, in the literature, there are barely any existing data on survival for patients with occult metastases compared with patients with positive lymph nodes detected preoperatively. The presence of occult metastasis could be a burdening factor requiring treatment escalation and a more aggressive follow-up than nodal disease detected via preoperative diagnostic imaging.

This study aimed to compare survival curves of patients suffering from lymph nodal metastases in a preoperatively N+ neck with those suffering from occult metastases. In addition, clinical characteristics of the primary tumor were analyzed to predict occult nodal disease.

## 2. Materials and Methods

### 2.1. Patient Cohort

This retrospective cohort study includes patients with an OSCC treated surgically with curative intent in our department between January 2010 and December 2016. Inclusion criteria were age older than 18 years, primary diagnosis of OSCC and follow-up of more than 60 months. Exclusion criteria were a history of other malignancy or radiation in the head and neck area or a history of chemotherapy or antibody therapy. In addition, all patients who had a resection status other than R0 were excluded.

This study was approved by the institutional review board of the University Hospital of Munich, Germany (Munich, Germany; UE 20-1096).

All available documents and data were analyzed: localization of the tumor, staging using the UICC system, grading, perineural invasion, lymphovascular invasion, venous invasion, tumor thickness and diameter, bone invasion, R status, number of resected lymph nodes, number of positive lymph nodes, localization of positive lymph nodes, occult metastases, diameter of metastases and localization of lymph nodal recurrence.

The radiological findings, as well as the decisions and documentation of the tumor conference, were reviewed concerning the suspicion of the presence of lymph node metastases.

The therapeutic concept for each patient consisted of OSCC resection within the appropriate safety margins, neck dissection and adjuvant treatment (radiation and/or chemotherapy) if necessary, as recommended in the German guidelines.

Our therapy was standardized in each patient and followed the guidelines applicable to us and the decisions of the interdisciplinary head and neck tumor conference. In each patient, the therapy consisted of resection of the OSCC, taking into account the respective safety margins and, if necessary, subsequent radio or radiochemotherapy depending on the stage. In case of positive N+ status or T3 and above, postoperative radiotherapy was recommended. Selective unilateral neck dissection of level I–III was performed. If the carcinoma was located in the midline or the tongue was affected, bilateral neck lymph node excision was performed. 

After completion of therapy, patients were followed up clinically every three months and radiologically through a contrast CT of the head and neck every six months for the first two years. After the second year, these intervals were adjusted to semi-annual clinical and annual radiological examinations. If local, locoregional or regional recurrence was clinically suspected, further restaging was performed as appropriate (CT, PET-CT, MRI). Salvage surgery was performed in case of recurrences. Follow-up without recurrence was considered complete after five years.

### 2.2. Statistical Analysis

Statistical analysis was conducted using R^®^ 24 version 1.3.1093 (Free Software Foundation, Boston, MA 02110-1335, USA). The Shapiro–Wilk test was used to determine the distribution pattern of the data, and they were not normally distributed. As survival analysis comparing patients with and without occult metastases and N+ necks to occult metastases, the Kaplan–Meier analysis was calculated. To detect potential risk factors for occult metastases, a logistic regression analysis was performed. Statistical significance was defined as *p* < 0.05. 

## 3. Results

### 3.1. Description of the Study Population

The patient cohort consisted of 226 patients (female *n* = 90, male *n* = 136; 64.1 years old (mean) ± 16.5 (SD) years) with primary diagnosed oral squamous cell carcinoma (OSCC). The majority of OSCCs were located on the floor of the mouth (*n* = 71/226, 31.4%) and the tongue (*n* = 70/226, 31.4%). The next common locations were the alveolar process of the mandible (*n* = 40/226, 17.6%) and the alveolar process of the maxilla and hard palate (*n* = 27/226, 11.9%). Other locations included the buccal mucosae and the intermaxillary region (19/226, 8.4%).

The carcinomas of patients with OM were mainly located in the tongue (*n* = 9/16) and the floor of the mouth (*n* = 4/16). The alveolar processes of the mandible (*n* = 2/16) and the maxilla (*n* = 1/16) were less numerous.

It was shown that 71.6% of patients were diagnosed with limited stage of disease (pT1 and pT2) and 28.4% with advanced stage of disease (pT3 and pT4). Cervical lymph node metastasis (pN1, pN2 and pN3) was histologically confirmed in 81 patients (35.8%). 

Histopathological grading showed that OSCCs were well differentiated (G1) in 41 of 226 patients (18.1%), intermediately differentiated (G2) in 148 of 226 patients (65.4%) (G2), and poorly differentiated (G3) in 37 of 226 patients (16.3%). In 80 of 226 patients (35.3%), the disease relapsed during the follow-up.

Occult metastases occurred in 16 of 226 patients. In 53 of 226 patients, neck lymph nodes were described as abnormal, altered, enlarged or malignant on CT imaging but had a pN0 neck after neck dissection.

Table 1 shows a significant correlation between grading and the occurrence of OM. OM occurs predominantly in T1 and T2 tumors (12 of 16).

The logistic regression with occult metastasis (yes vs. no) as the dependent variable showed that after adjustment, a higher tumor grading increased the chance of occurrence of occult metastasis 2.7-fold (OR (Odds Ratio) = 2.68, 95% CI (Confidence Interval): 1.07–6.7) (Table 2). Other factors had no significant influence on the occurrence of OM.

### 3.2. Survival Analysis

**Figure 1:**
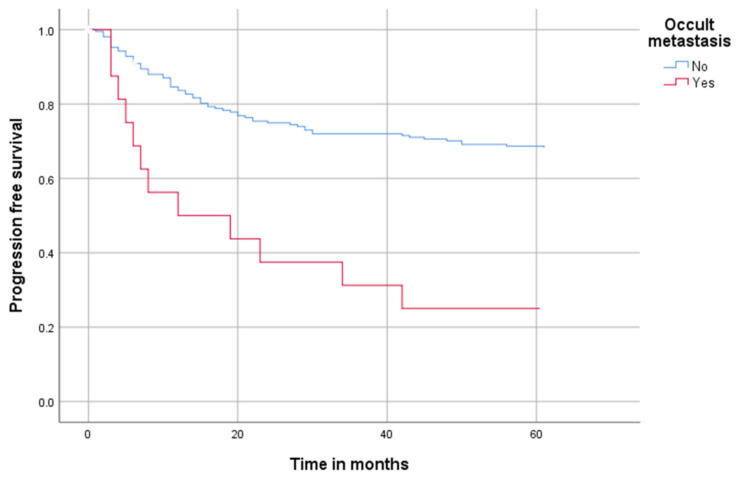
Progression-free-survival in all patients over 60 months; blue marks patients without occult metastasis (patients with N0 and N+ but previously diagnosed via staging; red marks patients with occult metastasis.

An analysis of progression-free survival using the Kaplan–Meier method is shown in Figure 1. Patients with OM had lower probabilities of progression-free survival. 

After 12 months of follow-up, 173 of 210 (82.3%) of the N0 patients were progression free. This reduced after 24 months to 155 of 210 (73.8%) and even further to 145 of 210 (69%) after 48 months. After 60 months, 141 of 210 were still progression free.

In the group with OM occurrence, after 12 months, 8 of 12 (66.6%) patients were progression free. After 24 months, 6 of 12 (50%) patients were still progression free. After 48 and 60 months, 4 of 12 (33.3%) patients were progression free.

**Figure 2:**
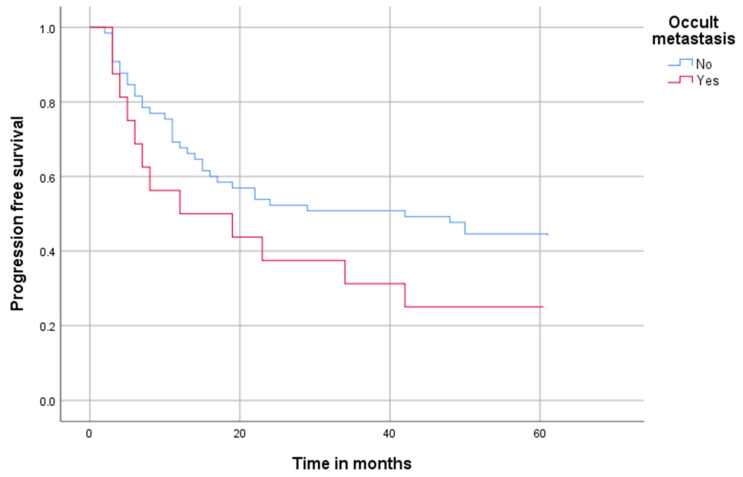
Progression-free survival in all N+ patients, occult metastasis yes vs. no. Progression-free-survival in all N+ patients over 60 months; blue marks patients without occult metastasis (but prior diagnosed metastasis via staging; red marks patients with occult metastasis.

After that, we analyzed the subgroup of N+ patients to see if patients with OM have different progression-free survival outcomes (Figure 2). Even within the N+ group, survival differentiates significantly.

Whereas in the N+ group without OM after 12 months, 53 of 65 (81%) were progression free, this reduced to 41 of 65 (63%) and 31 of 65 (47%) after 48 months. After 60 months, 28 of 65 (43%) were still progression free. 

In the Cox regression model, the predictors for progression-free survival were a positive N status and the occurrence of OM. A positive N status increased the risk of a negative outcome by 44% (HR = 1.44, 95% CI:1.08–1.93). The occurrence of OM increased the risk of a negative outcome by 133% (HR = 2.33, 95% CI:1.17–4.64). Other factors that might have had an influence, such as T classification, venous infiltration and grading, were not significant (Table 3).

## 4. Discussion

The occurrence of lymph node metastases continues to be a major challenge in OSCC. Occult metastases are a particular group of lymph node metastases, and these are associated with shortened survival and a higher risk of recurrence in the literature [20]. It has not yet been conclusively determined whether there are clinical or histologic factors that may favor the occurrence of occult metastases and may explain the increased risk for survival [16,20,21].

In our study, a relatively low rate of occult metastases of about 7% was found compared to the literature. This differs considerably from the data published by other groups, where the rate is closer to 20% [22]. Of course, several factors can lead to this. One reason could be the relatively high rate of lymph nodes described as enlarged/suspicious in the CT. Another cause could be the sensitive diagnostics and a detailed discussion of the imaging findings taking place in our clinic as part of the tumor board. Both would lead to a lower rate of occult metastases, as many questionable findings are more likely to be classified as malignant. In addition, it can be assumed that there has been progress in imaging in recent years, so findings that were previously below the detection limit may be more likely to become conspicuous.

Considering the yield of neck dissection, there is no difference between occult metastases and the other N+ or N0 patients. It can be assumed that the extent of neck dissection is independent of radiological assessment.

With regard to clinical and pathological parameters, a clear correlation with the occurrence of occult metastases is evident, especially in grading. Looking at the distribution of T stages, it appears that relatively many patients with occult metastases have a T1 or T2 stage. For other parameters, such as lymph vessel infiltration, our data show no correlation with the occurrence of OM [20,22,23].

Logistic regression showed an influence of grading on the occurrence of occult metastases. Other parameters show no correlations. In the literature, the histopathological parameter lymph vessel infiltration is regularly mentioned [24]. However, there was no clear correlation in our data. As a special marker, the lymphatic vessel density is sometimes assessed, which is displayed and evaluated by specific staining and includes counting the lymph vessels within the tumor. However, this is quite an extensive evaluation [21,25].

In the Kaplan–Meier survival analysis, patients with occult metastases had a significantly worse progression-free survival. Additionally, within the N+ group, occult metastases showed significantly worse survival than metastases previously detected on imaging.

The impact of occult metastases in univariate cox regression was significantly higher than that of normal N+ status. This is interesting because the histopathologic parameters do not necessarily explain why patients with occult metastases have shorter progression-free survival. In multivariate analysis, the presence of occult metastases is an independent prognostic factor for shortened survival.

One possible explanation is the constellation of low T stage and high grading in our cohort, such that many of these patients are patients with an aggressive tumor that was detected relatively early but had already formed micrometastases by this time.

If such patients are diagnosed later, they often have a more advanced stage of the disease, and CT scans can visualize the metastases. Another indication of a very aggressive course of the disease is that many patients already have several affected lymph nodes, i.e., synchronous metastasis. It is also possible that there was a bias concerning the potential risk due to the low T stage. However, this is contradicted by the fact that the yield rates within the N0, OM and N+ groups do not differ. Overall, such tumor operations are standardized to a maximum extent in our clinic to have little influence on progression-free survival.

According to the guideline valid for us, elective neck dissection is generally indicated from T1 onwards. However, there is still debate in the international literature on whether these cases benefit and whether neck dissection should be mandatory in T1 and T2 patients. Our data show that T1 and T2 tumors are at least as high risk as advanced tumors, even with an overall low rate of occult metastases [26,27]. Therefore, these data argue against foregoing cervical lymph node evacuation in the future for a radiological N0 neck.

One of the main weaknesses of this study is its retrospective nature. After such an extended period, decisions often cannot be discussed with the diagnosing radiologists to detect bias or misjudgment. It is also not possible to detect possible confounders. Prospective studies would offer the opportunity to record the disease’s course more precisely and discuss other possible causes of shortened survival.

Additionally, this study does not consider tumor markers such as CD36, which are associated with increased incidence of lymph node metastases.

## 5. Conclusions

The study conducted here shows that occult metastases lead to shorter progression-free survival compared to previously diagnosed metastases. High grading and a low T stage were more frequently associated with the occurrence of occult metastases. The data collected here argue against neck dissection in the case of a T1 or T2 tumor and a preoperative radiological N0 neck.

## Figures and Tables

**Table 1:** Patient characteristics.

Characteristic		N		Occult Metastasis			
				No (*n* = 210)		Yes (*n* = 16)	
Age in years		226		63.75 (SD ± 11)		70.33 (SD ± 8)	
Number of positive LNs		226		0.93		2.75	
Number of resected LNs		226		33.30 (SD ± 18)		30.75 (SD ± 15)	
Sex	Male	136	60%	126	93%	10	7%
	Female	90	40%	84	93%	6	7%
T classification	T1	94	42%	88	94%	6	6%
	T2	68	30%	62	91%	6	9%
	T3	20	9%	18	90%	2	10%
	T4	44	19%	42	95%	2	5%
N classification	N0	145	64%	145	100%	0	0%
	N1	25	11%	18	72%	7	28%
	N2	47	21%	39	83%	8	17%
	N3	9	4%	8	89%	1	11%
Grading	G1	41	18%	40	98%	1	2%
	G2	148	65%	139	94%	9	6%
	G3	36	16%	30	83%	6	17%
	G4	1	0%	1	100%	0	0%
Bone infiltration	0	186	82%	172	92%	14	8%
	1	40	18%	38	95%	2	5%
Perineural invasion	0	201	89%	188	94%	13	6%
	1	25	11%	22	88%	3	12%
Lymphovascular invasion	0	190	84%	179	94%	11	6%
	1	36	16%	31	86%	5	14%
Venous invasion	0	221	98%	206	93%	15	7%
	1	5	2%	4	80%	1	20%
Recurrence	No	146	65%	142	97%	4	3%
	Yes	80	35%	68	85%	12	15%

Association between the occurrence of occult metastasis and clinical and pathological parameters.

**Table 2:** Results of the logistic regression model.

Characteristic	OR (Odds Ratio)	95% CI (Confidence Interval)		Sig. (Significance)
		Lower	Upper	
T classification	0.815	0.397	1.672	0.577
Grading	2.684	1.075	6.703	0.035
Perineural invasion	1.532	0.328	7.163	0.588
Lymphovascular invasion	2.539	0.612	10.528	0.199
Venous invasion	1.153	0.088	15.033	0.913
Bone infiltration	0.477	0.059	3.875	0.489

**Table 3:** Cox regression model, predictors for progression-free survival.

Characteristic	OR (Odds Ratio)	95% CI (Confidence Interval)		Sig. (Significance)
		Lower	Upper	
Age	1.021	0.997	1.045	0.086
Sex	0.948	0.583	1.541	0.829
T classification	1.291	0.979	1.703	0.071
N classification	1.446	1.083	1.931	0.012
Grading	1.457	0.984	2.156	0.060
Perineural invasion	1.324	0.663	2.644	0.426
Lymphovascular invasion	1.162	0.603	2.240	0.654
Venous invasion	2.083	0.659	6.592	0.212
Bone infiltration	0.765	0.376	1.557	0.460
Occult metastasis	2.336	1.175	4.647	0.016

## Data Availability

Not applicable.
